# The role of inhibition in the processing of peripheral cues

**DOI:** 10.1007/s00426-024-02073-1

**Published:** 2025-01-06

**Authors:** Christian Büsel, Stephan F. Dahm, Pierre Sachse, Ulrich Ansorge

**Affiliations:** 1 Department of Psychology, University of Innsbruck, Innsbruck, Austria; 2 Institute of Cognition, Emotion, and Methods in Psychology, University of Vienna, Vienna, Austria; 3 University of Vienna, Vienna Cognitive Science Hub, Vienna, Austria; 4 University of Vienna, Research Platform Mediatised Lifeworlds, Vienna, Austria

## Abstract

The present study investigated the role of inhibition in peripheral cueing by nonpredictive cues. Based on past findings, we investigated the possibility that inhibition of learned irrelevant cue colors is typical of short cue-target intervals, with more competition for attention capture between cue versus target. In line with the expectation, in a modified contingent-capture protocol, with short cue-target intervals, we found same-location costs (SLCs) – that is, disadvantages for validly cued targets (cue = target position) compared to invalidly cued targets (cue ≠ target position) with consistently colored non-matching cues. In contrast, no such effects for inconsistently colored non-matching cues were observed with short intervals. In a control condition, with longer intervals, the differences between consistently and inconsistently colored cues were no longer observed. We argue that this effect is due to participants proactively inhibiting consistently colored non-matching cues with short intervals but not with long intervals, but that inhibition failed with inconsistently colored non-matching cues that could take on different possible colors. Alternative explanations in terms of object-updating costs or masking were ruled out. We conclude that the currently found type of inhibition of peripheral cues most likely reflected the limitation of proactively established control structures that could be used at the same time.

## Introduction

Everyday activity requires humans to interact with their environment in a meaningful way. However, the environment offers more sensory information than humans use or process. The implied selectivity can be seen as evidence of limited cognitive resources (e.g., Broadbent, 1958) or as a functional way to successfully work on relevant tasks (e.g., Ansorge et al., 2021). Regardless of the exact reasons for selection, the upshot is that only a subset of all potentially available information is used, meaning we need to understand the underlying mechanisms of such attentional selection for a variety of practical reasons, such as improving traffic safety (e.g., Grüner & Ansorge, 2017; Nikolic & Sarter, 2001) or optimized information placement (Gugerell et al., 2024; Scott et al., 2015).

Top-down selection of visual input is achieved either by facilitation of relevant input or by the inhibition of irrelevant input (cf. Folk et al., 1992; Gao & Theeuwes, 2020; Gaspelin & Luck, 2018; Noonan et al., 2018; van Morselaar & Slagter, 2020). In recent years, it became increasingly clear that active inhibition of irrelevant input supports visual attention (cf. Büsel et al., 2024; Forstinger et al., 2022; Kerzel & Barras, 2016; Wang & Theeuwes, 2018). At least three different principles of proactive (or offline) suppression – that is, suppression based on principles established before distractor onset – have been discussed (Noonan et al., 2018). First, distracting features can be inhibited by top-down processes directed at filtering out known irrelevant distractors based on explicit knowledge of the irrelevant features (e.g., Forstinger & Ansorge, 2024; Forstinger et al., 2022). Second, consistently used and, thus, also implicitly learned distracting features can be inhibited, which could be less flexible (e.g., Kerzel & Barras, 2016; Stilwell & Vecera, 2020). Third, focusing on relevant features can be inhibited by mutually inhibitory connections between various feature representations (e.g., Desimone & Duncan, 1995).

When looking at empirical results, however, in some cases, evidence for proactive inhibition seems to be missing entirely (e.g., Folk et al., 1992; Goller & Ansorge, 2015; Grubert & Eimer, 2016). There seem to be situations in which top-down selection is achieved exclusively by proactive facilitation of relevant input and without inhibition of irrelevant salient inputs at all. For example, in goal-directed selection in the *contingent-capture protocol* (Folk et al., 1992), subjects are asked to search for a target carrying a specific feature (e.g., blue). Shortly before the target search-display, salient singleton cues are presented either at the upcoming target position (valid trial) or away from it (invalid trial). The cues can either carry the task-relevant feature (here: blue; matching trial) or a task-irrelevant feature (e.g., red; non-matching trial). In addition, cues are usually spatially uninformative, meaning that the target is as likely to appear at the cued position as it is to appear at any other position. The common finding in this experimental protocol is that matching cues capture attention, whereas non-matching cues do not (Büsel et al., 2020). In this context, attention capture is indicated by response time (RT) validity effects (RT_invalid_ minus RT_valid_), suggesting that a matching cue captures attention to the correct (target) position in valid trials, but that attention has to be disengaged from a cued distractor position and redirected to the target in invalid trials. Importantly, no such validity effects are observed for non-matching cues (for a meta-analysis, see Büsel et al., 2020) which is indicative of simple ignorance rather than of proactive inhibition (e.g., Folk & Remington, 1998). Though some studies reported inverted validity effects or same-location costs (SLC) for non-matching cues (i.e., slower RTs in valid than in invalid trials; e.g., Büsel et al., 2019; Carmel & Lamy, 2014, 2015; Schoeberl et al., 2018) that could theoretically reflect proactive inhibition, research showed that at least some of these inverse validity effects were due to object-file updating costs rather than to proactive suppression of attention capture (cf. Carmel & Lamy, 2014, 2015): Cues and targets presented successively and at the same location (i.e., in valid trials) are represented as a single perceptual object. In case a matching cue precedes the target at the same location, the object-file needs no revision or updating because the task-relevant feature, for example the color, of cue and target is the same. However, if the target appears at a location that was previously occupied by a non-matching cue, the same task-relevant feature, for example, the color, needs to be updated from cueing to target display. Obviously, such updating costs under non-matching conditions would only occur in valid trials but not in invalid trials, meaning that these object-updating costs (REF) could also lead to an SLC that has nothing to do with the suppression of attentional capture.

Yet, when using a probe task, Büsel et al. (2024; see also Gaspelin et al., 2015) observed that participants’ report of probes presented instead of the visual search target displays was suppressed for probes at locations cued by cues consistently colored in a non-matching color. This suppression was found relative to cues with the task-relevant color. However, the same suppression relative to probes at the matching cues’ position was not found for inconsistently colored non-matching cues. Note that under these conditions, object-updating costs could not account for the SLCs of the non-matching cues because consistently colored and inconsistently colored cues were both of a different color than the target and, thus, object-updating costs would have been the same under both these non-matching cueing conditions. The authors therefore concluded that proactive inhibition of consistently colored non-matching cues might contribute to their lower validity effect than of matching cues. This form of proactive suppression, based on past experience with a consistent color cue, most likely reflected a type of experience-based, learned distractor suppression (cf. Kerzel & Barras, 2016; Noonan et al., 2018).

However, if inhibition of consistently colored non-matching cues holds, why is this inhibitory processing of non-matching cues not regularly observed as an SLC in the contingent-capture protocol? One potentially decisive procedural difference between those protocols that yielded evidence for proactive inhibition of consistently colored non-matching cues (Büsel et al., 2024) and those protocols that do indicate simple ignorance of named cues (e.g., Folk & Remington, 1998) is the stimulus-onset asynchrony (SOA) between cue and probe (in Büsel et al., 2024) or cue and target (in typical attention-capture experiments; cf. Folk & Remington, 1998). The typical SOA in contingent-capture studies is at least 150 ms, but evidence for proactive inhibition of consistently colored non-matching cues was found with a much shorter interval of 0 ms between cue and probe in Büsel et al. (2024; see also Tassinari et al., 1994, for related evidence in form of an SLC in a more standard cueing protocol).

In the present study, we therefore tested if more evidence for proactive inhibition could be found with consistently colored non-matching cues presented with a shorter than with a longer SOA prior to the target. For example, direct competition between non-predictive, irrelevant and non-matching cues and targets should be stronger with a short than with a long interval between cue (or distractor) and target (cf. Pomper et al., 2023). Thus, participants could use proactive suppression of known distracting features (here, consistently colored non-matching cue) on top of facilitation of relevant features only in the more competitive, shorter SOA conditions, but not or less so in the less competitive, longer SOA condition. This would be in line with the observation that proactive inhibition is more typical of difficult tasks (cf. Conci et al., 2019). This would also explain why studies using electroencephalography failed to show much evidence of proactive inhibition of (consistently colored) non-matching cues in form of a distractor positivity (Hickey et al., 2009; Szaszkó et al., 2024). Therefore, to separate cue-elicited from target-elicited event-related potentials, longer cue-target SOAs are used (e.g., Ansorge et al., 2011; Eimer & Kiss, 2008).

**Fig. 1:**
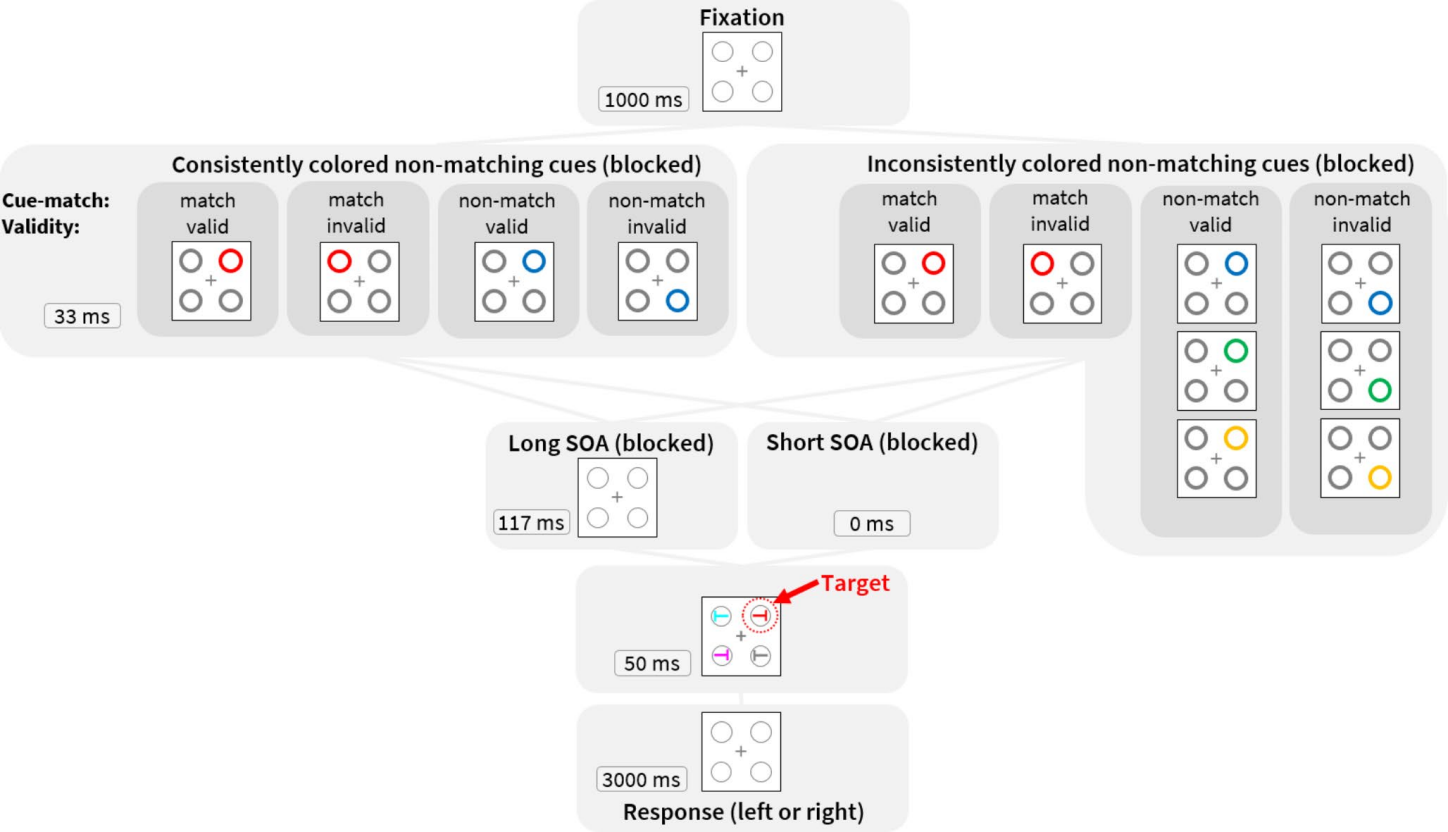
Examples of trials in 150 ms and 33 ms cue-target stimulus onset asynchrony (SOA) conditions. In every trial, only one cue was presented. *Cue-match* denotes whether the cue had the same color as the searched-for target (match) or not (non-match), whereas *Validity* refers to the cue’s location relative to the target’s location (i.e., valid = same position; invalid = different position)

To test the predictions, we conducted an experiment in which consistently and inconsistently colored non-matching cues were presented prior to targets in one half of the trials (see Fig. 1). Here, color consistency of the non-matching cues was a blocked factor. In the other half of the trials of both blocks, cues matching the searched-for target color were presented. In one block of trials of both the consistent and the inconsistent condition, a short cue-target SOA was used. Here, under these conditions of enhanced cue-target competition, we expected participants to try and inhibit cues, but this should be easier (or even only possible) for cues with a known (learned) color than for cues of a changing color (cf. Büsel et al., 2024; Kerzel & Barras, 2016). Therefore, we expected SLCs for consistently colored non-matching cues but less so (or maybe even not at all) for inconsistently colored non-matching cues. In other words, the inconsistently colored non-matching cues served as an important control condition to account for potential forward masking effects of differently colored cues on target visibility, which might otherwise also explain an SLC in short cue-target interval conditions (e.g., Lupiáñez & Weaver, 1998). Just as object-updating costs, forward masking – that is, a diminution of target-color perception by a differently colored preceding cue at the same location – would have been the same in consistently colored and inconsistently colored non-matching cueing conditions.

In another block of trials of both consistent and inconsistent conditions, a more typical, longer cue-target SOA was employed. Here, we expected less need for inhibition as the competition for attention between cue and target was not that fierce and, thus, the more conventional finding of no evidence for SLCs whatsoever by both types of non-matching cues, regardless of their color consistency.

To note, in the consistently colored non-matching conditions, the predictions concerning the relationship between SOAs and SLCs were the opposite to that based on object-file updating costs: Object-file updating costs require time to build up (cf. Carmel & Lamy, 2015). When a cue-target SOA of 50 ms was used, object-file updating costs were absent. Only when a cue-target SOA of 150 ms was used, object-file updating costs in the form of SLCs were found. Object-file updating costs should, thus, lead to an SLC with long SOAs, but not with short SOAs (Carmel & Lamy, 2015). In addition, with a long SOA, SLCs based on object-updating costs were expected for known and well learned (or consistent) as well as for less well learned (or inconsistent) non-matching cues. The same logic applies to forward masking effects: These would be stronger with short intervals than long intervals (cf. Kafaligönül et al., 2009). Finally, besides the non-matching cues, we used matching cues as a further control condition. The matching cues were expected to yield regular and relatively similar validity effects under both SOA conditions – that is, advantages in valid compared to invalid conditions.

Finally, we also looked into the development of potential SLC across time under short-interval/consistent conditions, to understand the origin of the effect. First, it could be that the SLC reflected a rational strategy to prevent bottom-up capture by irrelevant singletons (e.g., Gaspelin et al., 2015). In that case, it was to be expected that participants first had to get to know the cue’s consistent color to build up their top-down control setting to suppress the known color. If this was the case, we would have expected the SLC to grow across time and experience with the consistently colored non-matching cue. Note, however, that the cueing or validity effect in the RTs of the inconsistently colored short-interval/non-matching conditions was largely missing. Hence, at least in the RTs, there was not much bottom-up capture to be prevented by proactive suppression. Therefore, secondly, it is also possible that proactive suppression of a consistently colored and misleading (i.e., uninformative) singleton cue is mostly a prophylactic but ultimately unnecessary strategy. If this was the case, the SLCs could even be stronger relatively early in the experiment and diminish over time, when participants experience that proactive inhibition is not really required. For instance, participants’ shielding settings of proactive and feature-specific suppression against distraction by the consistently colored non-matching cues could sometimes inadvertently be down. This could be due to lapses in attentional control, for example. In these trials, participants might not experience much interference by the non-matching cue despite not having felt prepared well to suppress the cue. If this was the case, participants would have learned that their prophylactic suppression of the known color of the consistently colored non-matching cues was in fact irrational and nothing to waste much energy upon. In fact, proactive suppression might be more resource-consuming and, thus, a waste of energy where it is not necessary (cf. Rajsic et al., 2020). Thus, we also might find the exact opposite pattern: more evidence of SLCs based on quickly acquired cue-color knowledge early in the short-interval/consistently colored non-matching conditions but less suppression and SLCs or none at all later in these conditions.

## Methods

### Participants

Seventy-two participants (47 female; *M*_age_ = 22.2 years, *SD*_age_ = 2.8) were tested in the present experiment. Participants were students of the University of Innsbruck, gave informed consent, and received course credit for their participation. We derived the necessary sample size from the observed difference in validity effects of consistently and inconsistently colored non-matching cues under 0 ms cue-probe SOA conditions in Büsel et al.’s (2024) Experiment 1. These authors observed validity effects by inconsistently colored non-matching cues that were 19 ms larger than those by consistently colored non-matching cues, *t*(38) = 2.21, *p* = .033, *d* = 0.51. In order to detect a difference of the same size with a power of 95% (two-sided), we would have needed 51 participants. However, due to unstandardized testing conditions, we opted to test more participants to balance out any potential influences of testing environments.

### Apparatus

The online experiment was created using OpenSesame (Mathôt et al., 2012) and hosted on a JATOS server (Lange et al., 2015).

### Stimuli

Exact stimulus sizes are not known due to the online nature of this experiment. The degrees of visual angle reported here stem from a screen resolution of 1,280 × 720 px and an assumed viewing distance of 57 cm. All stimuli were presented against a black background. Four gray (RGB: 128, 128, 128) circles (diameter: 2°; line thickness: 0.1°) surrounding a central fixation cross (0.5 × 0.5°) were presented in the fixation display. The circles were placed at the corners of an imaginary square with a side length of 6°. In the cueing display, the circles thickened (0.4°) with one circle constituting a color singleton and the remaining three circles gray non-singletons. In the target display, four colored *T*s (1.1 × 0.9°) were presented, one at the center of each circle. Two *T*s were rotated 90° to the left, and two were rotated 90° to the right. One of the *T*s was colored in the searched-for target color (red; 140, 0, 0), with the remaining three *T*s being colored cyan (0, 65, 65), magenta (90, 50, 90), and gray. This ensured that participants engaged in feature-search mode, rather than simply searching for uniqueness (cf. Bacon & Egeth, 1994). Under the consistent non-matching cue color conditions, the non-matching cue was consistently colored blue (0, 0, 255). In the inconsistent block, the non-matching cue was randomly either blue, green (0, 128, 0), or yellow (255, 255, 0), with the cue color being unpredictable from trial to trial. In all blocks, the matching cue was always of the same (red) color as the target.

### Design and procedure

Example trials can be seen in Fig. 1. At the beginning of each trial, participants were presented with the fixation display for 1 s. The cueing display was shown for 33 ms both under 33 ms and 150 ms cue-target SOA conditions^1^. In the 33 ms cue-target SOA condition, the target-search display immediately followed the cueing display, whereas in the 150 ms cue-target SOA condition the fixation display was presented for 117 ms between cueing and target-search display. The target-search display remained visible for 50 ms. Participants were asked to indicate the orientation of the red *T* as quickly and accurately as possible on their keyboards by pressing either the y-key (*T* rotated counter-clockwise) or the m-key (*T* rotated clockwise). If no response was given within 3 s, the experiment proceeded with the next trial. In case of a wrong response or none at all, feedback ‘Falsch’ (‘Wrong’) was provided for 500 ms. Each block started with 20 practice trials which were excluded from analyses.

Cues were not predictive of the target location or of the orientation of the target *T*. Cues and targets were equally likely presented at each location. Across trials, cue and target positions were uncorrelated, leading to 25% valid and 75% invalid trials. Cues were equally likely matching or non-matching. In inconsistently colored non-matching cue conditions, each of the three possible cue colors was equally likely.

The experiment consisted of four blocks, the order of which was counter-balanced across participants. Each block consisted of 200 trials. While cue validity (valid, invalid) and cue match (matching, non-matching) were randomized within each block, cue-target SOA (33 ms, 150 ms)^1^ and non-matching cue consistency (consistent, inconsistent) were varied between blocks. Participants completed 20 practice trials before each block, which were subsequently not analyzed. After every 50 trials, participants could take a self-paced break. Altogether, the experiment took about 45 min.

## Results

RTs deviating more than 2.5 *SD*s from participants’ respective condition were removed, leading to a data loss of 2.2%. Furthermore, one participant was removed due to below chance accuracy. Only correct trials were included in RT analyses.

### Response times

Correct RTs were fed into a repeated-measurements analysis of variance (ANOVA), with the independent variables Validity (valid, invalid), Cue-match (matching, non-matching), Non-matching cue consistency (consistent, inconsistent), and SOA (33 ms, 150 ms). A main effect was found for Validity, *F*(1, 70) = 411.48, *p* < .001, η_p_^2^ = 0.85. Furthermore, the interaction between Cue-Match and Validity was significant, *F*(1, 70) = 415.13, *p* < .001, η_p_^2^ = 0.86. Additionally, all three-way interactions reached significance: Non-Matching Cue Consistency × Validity × Cue-Match, *F*(1, 70) = 6.44, *p* = .013, η_p_^2^ = 0.08, Non-Matching Cue Consistency × Validity × SOA, *F*(1, 70) = 6.40, *p* = .014, η_p_^2^ = 0.08, Non-Matching Cue Consistency × Cue-Match × SOA, *F*(1, 70) = 8.35, *p* = .005, η_p_^2^ = 0.11, and Validity × Cue-Match × SOA, *F*(1, 70) = 16.00, *p* < .001, η_p_^2^ = 0.19.

**Fig. 2:**
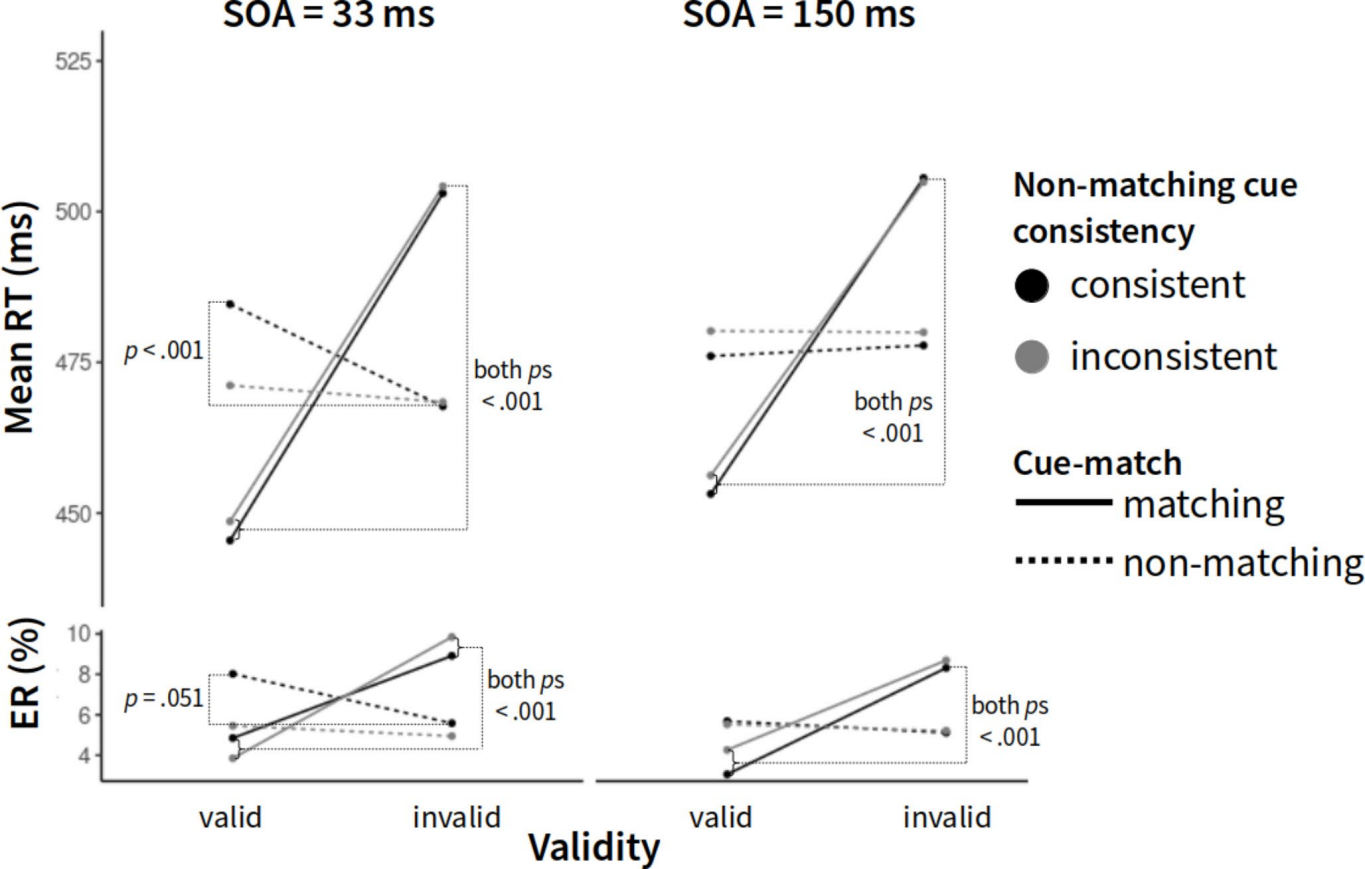
Mean response times (RTs; y-axis) as a function of validity (x-axis), stimulus-onset asynchrony (SOA; left = 33 ms, right = 150 ms), non-matching cue consistency (line colors), and cue match (line types)

All variables also entered a significant four-way interaction, *F*(1, 70) = 4.20, *p* = .044, η_p_^2^ = 0.06 (Fig. 2). First, looking at the 150-ms SOA condition, we found validity effects for matching cues in both blocks with consistent non-matching cues, *M*_ValidityEffect_ = 52 ms, *t*(70) = 16.52, *p* < .001, *d* = 0.86, and in blocks with inconsistent non-matching cues, *M*_ValidityEffect_ = 49 ms, *t*(70) = 13.89, *p* < .001, *d* = 0.79. Non-matching cues did not capture attention, regardless of cue color consistency (*p*s > 0.56). With the shorter 33-ms SOA, this pattern was more intricate: Matching cues, again, captured attention both in blocks with consistent, *M*_ValidityEffect_ = 58 ms, *t*(70) = 17.74, *p* < .001, *d* = 1.13, and in blocks with inconsistent non-matching cues, *M*_ValidityEffect_ = 56 ms, *t*(70) = 18.25, *p* < .001, *d* = 1.05. Consistent non-matching cues, however, led to a significant inverted validity effect, *M*_ValidityEffect_ = − 17 ms, *t*(70) = − 6.8, *p* < .001, *d* = 0.34, meaning responses were faster in invalid than in valid conditions. Inconsistent non-matching cues did not elicit such an inverted validity effect or any significant validity effect (*M*_ValidityEffect_ = − 3 ms; *p* = .26, *d* = 0.05).^2^

### Error rates

An identical analysis with arc-sine transformed error rates (ERs) yielded main effects for Validity, *F*(1, 70) = 156.15, *p* < .001, η_p_^2^ = 0.69, and SOA, *F*(1, 70) = 9.68, *p* < .01, η_p_^2^ = 0.12. Participants committed more errors in invalid (7.1%) than valid trials (5.1%). They also committed slightly more errors in 33-ms SOA blocks (6.4%) than in 150-ms SOA blocks (5.7%). Three two-way interactions were found to be significant as well, with an interaction between Validity and Cue-Match, *F*(1, 70) = 106.57, *p* < .001, η_p_^2^ = 0.60, Cue-Match and Non-Matching Cue Consistency, *F*(1, 70) = 4.56, *p* = .036, η_p_^2^ = 0.06, and Non-Matching Cue Consistency and SOA, *F*(1, 70) = 156.15, *p* = .005, η_p_^2^ = 0.11. Since non-matching cue consistency and SOA entered a three-way interaction with validity, we more closely examined the first two two-way interactions. The two-way interaction between Validity and Cue-Match was due to a validity effect for matching cues (valid: 4% vs. invalid: 8.9%), *t*(70) = 15.41, *p* < .001, *d* = 1.5, and no meaningful difference between valid and invalid trials for non-matching cues (*p* = .7). The two-way interaction between cue match and non-matching cue consistency was characterized by an increased error rate (6.7%) following matching cues compared to non-matching cues (5.3%) in blocks with inconsistent non-matching cues, *t*(70) = 2.28, *p* = .025, *d* = 0.22. No such difference was found in consistent non-matching cue blocks.

Regarding the three-way interaction, we found significant validity effects under 150-ms SOA conditions, both in blocks with consistent (valid: 4.4% vs. invalid: 6.7%), *t*(70) = 9.31, *p* < .001, *d* = 0.9, and in blocks with inconsistent non-matching cue conditions (valid: 4.9% vs. invalid: 7%), *t*(70) = 6.88, *p* < .001, *d* = 0.71. A fairly similar pattern was found under 33-ms SOA conditions: a small, yet reliable validity effect was found in blocks with consistent non-matching cue conditions (valid: 6.4% vs. invalid: 7.2%), *t*(70) = 3.59, *p* < .001, *d* = 0.37, and a larger validity effect was found in blocks with inconsistent non-matching cue conditions (valid: 4.7% vs. invalid: 7.4%), *t*(70) = 7.9, *p* < .001, *d* = 0.87.

### Origins of same-location costs

**Fig. 3:**
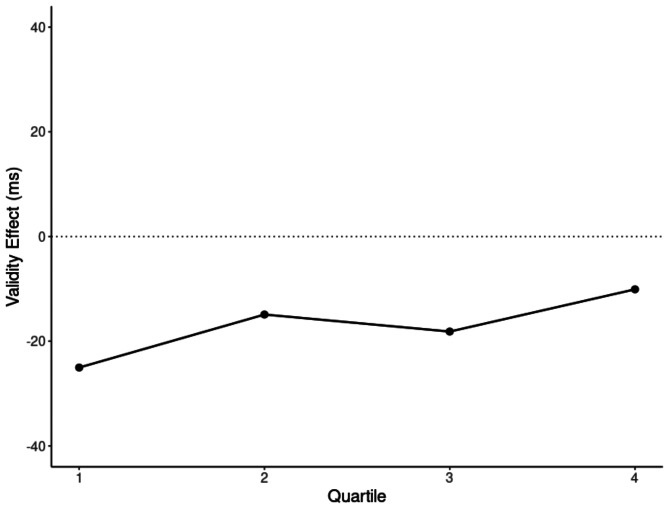
Development of same-location costs (SLCs) in consistent short SOA blocks. SLCs tend to decrease over the course of the block

A visual inspection of Fig. 3 suggests that subjects shave learned the consistent non-matching cue color very early on. However, participants seemed to have increasingly stopped using the consistent non-matching cue color as a negative template for suppression (Rajsic et al., 2020). This interpretation is only tentative, however, as a repeated-measurements ANOVA with the factors Validity (valid, invalid) and Quartile (1st, 2nd, 3rd, and 4th quartile) only resulted in a significant main effect of validity, *F*(1, 70) = 46.90, *p* < .001, η_p_^2^ = 0.40, which was due to an overall SLC of 17 ms. Neither the main effect of, nor the interaction with quartile was significant (both *p*s > 0.23).

## Discussion

In the present experiment, we examined whether participants proactively inhibit irrelevant, non-matching cues once these cues are consistently presented in one and the same color and the temporal interval between cue and target was short enough to increase competition of cues and targets for the capture of attention. This expectation was confirmed. Non-matching cues elicited an SLC – slower responses in valid than invalid trials – only if they were shown consistently in one and the same color and with a relatively short SOA prior to the target. In contrast, non-matching cues presented in an inconsistent color or with a longer cue-target interval were not actively suppressed, as is typically observed in contingent-capture research (cf. Ansorge et al., 2011; Folk & Remington, 1998; for a review, see Büsel et al., 2020).

These observations are in line with the assumption that, once participants learn a consistent color of a non-predictive and non-matching cue, participants can proactively initiate inhibition of these cues so that such non-matching cues elicit inhibition that then spills over to targets at cued positions and delays successful search for the validly cued targets. At the same time, the data pattern rules out that SLCs of the consistent non-matching cues were better explained by masking or by object-updating costs. Masking and object-updating costs should have negatively affected processing of all validly cued targets where the preceding non-matching cue is of a different color than the target and the cue-target interval is short (Lupiáñez & Weaver, 1998). However, the inconsistently colored non-matching cues did not elicit an SLC. Object-updating costs would have also been mostly restricted to long-SOAs, and they should have been absent at short SOAs below 150 ms, such as ours of 33 ms (cf. Carmel & Lamy, 2015). However, SLCs were restricted to short SOAs and missing with long SOAs. They were also restricted to consistently colored non-matching cues with short SOAs, and they were missing with inconsistently colored non-matching cues in both SOA conditions.

We argue that proactive inhibition was most likely triggered through an increased competition between attention capture by the targets versus the cues under short SOA conditions. However, why should this be if attention capture can be successfully restricted to the most relevant features with a bit more time between nonpredictive cue and target? A standard answer to this question would be that the singleton cues were of high salience and, thus, captured attention in a bottom-up way, even in non-matching conditions (cf. Theeuwes, 1992, 2010; Theeuwes et al., 2000). However, during the cue-target interval, attention is then disengaged from the cue location and prior to target occurrence, unless a more difficult cue-target discrimination prevents this from happening in the matching cue conditions. Yet, ERP studies are not in line with this assumption (e.g., Ansorge et al., 2011). In these studies, there is simply not sufficient evidence for the initial capture of attention by just any salient singleton cue, be it task-relevant or task-irrelevant.

Therefore, we believe that instead the weighting of salience, as one influence on attention capture, is itself subject to extraneous influences such as the (temporal) discriminability of relevant targets between irrelevant distractors (here: cues) – that is, to the signal-to-noise ratio in the displays (cf. Duncan & Humphreys, 1989). This is related to the load theory (Lavie, 2005; Lavie & Tsal, 1994) and the priority accumulation framework (Yaron & Lamy, 2021). According to load theory, the cognitive demands imposed by the task at hand can increase bottom-up influences on attention, and we would argue that lower signal-to-noise ratios in the input can increase these demands. According to the priority accumulation framework, cue-based influences on the priority of target and distractor representations in priority maps increase with perceptual difficulty, and, again, perceptual difficulty would be higher under low than high signal-to-noise ratio conditions (corresponding to short- vs. long-SOA conditions in the present study).

Assuming that participants’ proactive inhibition was based on prior experience with or learning of the irrelevant color of the cue, it is easy to understand that evidence for inhibition was restricted to consistently colored non-matching cues. There was simply not the same opportunity to learn the many different non-matching colors or to successfully inhibit all of them at the same time with inconsistent non-matching cues and with matching cues that are consistent with top-down search strategies. For example, it might be difficult to use top-down sets of attentional control for more than one or two features (e.g., one positive target color and one negative, i.e., to-be-suppressed, cue color) at the same time (cf. Kerzel & Grubert, 2022). If this is the case, the proactive inhibition of several potential non-matching cue colors in the inconsistent conditions would have suffered. Likewise, it might be more difficult to use one and the same feature (here, the color of the target that was also used for the matching cue) for the almost simultaneous facilitation of the processing of this feature (here, as a searched-for target color) and its suppression (here, as a nonpredictive matching cue’s color). However, one might wonder whether the current study indeed provides more evidence of stimulus-driven or bottom-up attention capture by all salient singleton cues in general under short SOAs than long SOAs. After all, if the cues created more competition for attention capture under short SOAs than long SOAs, capture effects of the cues should be larger under short SOAs than long SOAs of the present study because participants could not have prevented more stimulus-driven capture of attention by two out of three cue types (i.e., inconsistently colored non-matching cues and, equally salient, top-down matching cues) with short SOAs than with long SOAs. In fact, in the present study, this expectable higher stimulus-driven capture effect by just any singleton cue was found in the form of a stronger validity effect in ERs with short SOAs for blocks with inconsistently colored non-matching cues in comparison with blocks of consistently colored non-matching cues. In contrast, the same difference between validity effects in error rates in consistent and inconsistent blocks was not significant with a longer cue-target SOA, and the error rate with long SOAs was generally lower with the longer than with short SOAs.

An alternative explanation of the central finding of the SLC in terms of deallocation and without recourse to higher competition under short- than long SOA conditions is less likely. According to the deallocation account, we would have expected to see more evidence of stimulus-driven capture under the short SOA than under the long SOA condition because in the latter conditions, by the time that the target was shown, attention might have been more likely deallocated from the cue and back to a more neutral location (cf. Theeuwes et al., 2000). However, if this explanation were true, a cue-elicited capture effect should be seen in ERPs, but this capture effect is typically not observed (e.g., Ansorge et al., 2011).

This brings us to a final point of the discussion: the likely mechanism of inhibition. We assumed that inhibition was proactive, in the sense that inhibition relies on some type of offline representation that participants set up prior to the to-be-suppressed stimulus. We think that the assumption is justified in light of prior findings on reactive (rather than proactive control) that suggested that the time between suppressed distractors and searched-for targets needed to be at least more than 200 ms for reactive control to take effect (cf. Moher & Egeth, 2012). With the current short SOA of 33 ms, however, the time to suppress the cue colors in a reactive way and before target onset would have been too short for such reactive suppression. We also assumed that, in the current study, proactive inhibition depended on prior experience with the to-be-suppressed features (here, the cue colors). In line with this assumption, the more exposure our participants had with a non-matching color, the more evidence for suppression was found. The finding that proactive inhibition can be based on learned distractor features is in line with prior research confirming this principle (Noonan et al., 2018; Stilwell & Vecera, 2020). However, we do not deny that other principles of proactive inhibition might be at work depending on the exact side conditions (cf. Forstinger et al., 2022).

A potential limitation of the current study is that it was conducted online, without the stringent control of ecological factors. While this may have introduced some noise into our data, previous research suggests that sufficiently powered online studies provide similar results as lab-based studies (Frătescu et al., 2019). For example, the contingent-capture effect has been repeatedly replicated (e.g., Büsel et al., 2024; Experiment 2 in Büsel et al., 2021), leading us to believe that our study was sufficiently powered to allow for precise conclusions. Nonetheless, a replication of our findings in a lab-based, well-controlled study is desirable.

In conclusion, our results neatly integrate with several lines of research on (learned) suppression (Geyer et al., 2008; Gaspelin & Luck, 2018; Kerzel & Barras, 2016; Pomper & Ansorge, 2021; Stilwell & Vecera, 2020; Wang & Theeuwes, 2018; Won et al., 2019), suggesting that SLC and suppression are limited to a single feature or maybe two features at best. Our findings might, thus, also generally be more in line with the observation that currently active top-down sets (here: for proactive suppression) could be limited to one or two (cf. Büsel et al., 2019; Kerzel & Witzel, 2019; Olivers et al., 2011), and that proactive sets for more than two to-be-suppressed features were too difficult to maintain in working memory.

## Data Availability

The original data can be found at https://osf.io/74w9z/.

## Footnotes

1 Note that participants may have used computer monitors with varying screen refresh rates. Here we report (rounded) presentation durations assuming that most participants used monitors with a conventional 60 Hz refresh rate (i.e., possible presentation durations in increments of 16.6 ms).

2 One potential shortcoming of our study was the persistent use of blue non-matching cues in consistent non-matching cue conditions. In order to exclude any potential color dependent influences on the results in the inconsistent non-matching cue conditions, such as less average masking by the non-matching color and non-blue singleton cues, we re-ran the analyses with only trials with blue cues in the inconsistent non-matching condition. Again, no SLCs were observed under 33 ms or 150 ms SOA conditions (both *p*s > 0.69). Additionally, we investigated whether validity in previous trials modulated validity effects, as has been shown by Goller and Ansorge (2015) and Qian et al. (2022), which was not the case in our experiment. We thank the reviewers for raising these possibilities.
